# The Association of Residence Permits on Utilization of Health Care Services by Migrant Workers in China

**DOI:** 10.3390/ijerph18189623

**Published:** 2021-09-13

**Authors:** Haochuan Xu, Han Yang, Hui Wang, Xuefeng Li

**Affiliations:** 1 West Center for Economic Research, Southwestern University of Finance and Economics, Chengdu 611130, China; xuhaochuan@163.com (H.X.); fzyanghan@126.com (H.Y.); lixuefeng@swufe.edu.cn (X.L.); 2 School of Accounting, Southwestern University of Finance and Economics, Chengdu 611130, China

**Keywords:** residence permit, health care services, migrant worker

## Abstract

Due to the limitations in the verifiability of individual identity, migrant workers have encountered some obstacles in access to public health care services. Residence permits issued by the Chinese government are a solution to address the health care access inequality faced by migrant workers. In principle, migrant workers with residence permits have similar rights as urban locals. However, the validity of residence permits is still controversial. This study aimed to examine the impact of residence permits on public health care services. Data were taken from the China Migrants Dynamic Survey (CMDS). Our results showed that the utilization of health care services of migrant workers with residence permits was significantly better than others. However, although statistically significant, the substantive significance is modest. In addition, megacities had significant negative moderating effects between residence permits and health care services utilization. Our research results emphasized that reforms of the household registration system, taking the residence permit system as a breakthrough, cannot wholly address the health care access inequality in China. For developing countries with uneven regional development, the health care access inequality faced by migrant workers is a structural issue.

## 1. Introduction

Migrant workers (international and internal) usually refers to workers who are employed outside their original residence. Many migrant workers work in dangerous and exploited environments, and they might be exposed to considerable hazards and health risks [1]. Without local identification, many migrant workers are unable to receive the same health care services as locals in the workplace, resulting in health care access inequality [2]. Considering the bidirectional contribution of migrant workers to their destination and original residence, maintaining their health is highly beneficial [3]. Whether from the perspective of human rights or interests, it is of considerable significance to tackle health care access inequality. However, even in countries with high Universal Health Coverage (UHC) scores, there are still enormous challenges in addressing the inequality in access to health care for some migrant workers [4,5,6]. To achieve UHC, some countries provide visas for migrant workers, aiming to solve the problem of migrants’ lack of local identification and ensure their fair access to public health care services [7,8].

In the past decades, urban migration had been particularly prevalent in China. In the Chinese context, “migrant workers” generally refers to the labor force groups who work as temporary residents in non-registered locations without changing their household registration. The household registration system is a system of population registration [9]. In China, citizens are registered according to the administrative region of their birthplace. Plenty of migrant workers flow from rural areas to cities or medium and small-sized cities to large cities [10]. These migrant workers play an essential role in China’s urbanization and industrialization [11,12]. However, the large-scale migration of the population might break the original pattern of interregional interests. The administrative resources, the proportion of financial transfer payment, and land quota allocation are still determined by the registered residence population’s size. The large influx of population makes the inflow areas undertake more public service responsibilities, which increases the pressure on public services and may lead to a situation where the supply cannot meet the demand [13]. Under the household registration system restrictions, many migrant workers temporarily live in working cities and are treated unequally when using public services [14,15,16]. For example, it is difficult for migrant workers to enjoy the same quality of urban public welfare programs as local residents in housing, health care, and education, which aggravates their living burden in these areas [17,18,19]. Besides, most migrant workers are engaged in low-skill jobs [20], and their contract signing rate is far lower than the national level [21]. The unstable working conditions pose a challenge to the sustainability of their income. In response to potential risks, they try to reduce spending and increase savings [22]. Many migrant workers maintain a low standard of living in the city and take the strategy of making money in the city and returning to their hometown for consumption or investment [23,24]. Based on the above reasons, migrant workers can be regarded as living on the “edge of the city”. Living in an unstable environment lacking family support, public infrastructure, and social support networks, migrant workers lack the necessary health protection and face unnoticed high health risks [25,26]. This problem not only threatens the health of migrant workers but also affects public health in cities.

The Chinese government has repeatedly promised to speed up the household registration system’s reform to accommodate more migrant workers [27]. Among various reform policies, residence permits play an important role. The intended functioning of the residence permit was outlined in the Interim Regulations on Residence Permits, which was implemented nationwide on 1 January 2016. Functionally, the residence permit is similar to the “visa”. For most cities, if migrant workers meet one of the three conditions of stable employment, stable residence, and continuing study, they can apply for a residence permit in this city. Although the functioning of residence permits in each city may be different, the stated purpose is the same. Through their residence permits, migrant workers have a guaranteed right to use public services, including health care services. Taking the reimbursement process of medical expenses as an example, medical cost is one of the important factors that cause inequality in medical services utilization. High costs of health care have resulted in migrants’ use of unsupervised self-treatment or substandard care [28,29], which put them at risk of poorer health outcomes than local urbanites [30]. The Chinese government has tried to reduce medical expenses by popularizing social health insurance, but many migrant workers only have insurance in their hometowns. For migrant workers whose insurance relationship is not local, they need to return to their hometown to reimburse their expenses through a complicated process. Therefore, some scholars believe that medical insurance is not a decisive factor in medical services utilization for migrant workers [31,32]. During the reimbursement process for migrant workers, it is necessary to provide proof of residence in the city where the medical expenses are incurred. The residence permit is a valid proof of residence, which ensures that migrant workers can be reimbursed for medical expenses conveniently. Besides, the residence permit also empowers migrant workers to apply for local social health insurance, so that they can directly apply for reimbursement locally.

By August 2017, 29 provincial units of China had implemented points systems for migrants, and over 43 million migrants had received their residence permits [33]. However, the actual effect of the residence permit is still controversial and poorly understood. Prior studies found that public service standards differed between residence permits and local household registration, which is far from the stated purpose. Taking education services as an example, although many cities give children of migrant workers with residence permits access to public schools, the quota is limited [13]. Furthermore, due to the high population density in Chinese cities and the shortage of educational resources, there are significant differences in the quality of schools. The access opportunities of high-quality public schools are closely related to housing ownership in high-quality school districts [34]. Many studies have shown that the quality of education services has a significant impact on housing prices [35,36,37,38]. Considering that most migrant workers are engaged in low-skilled jobs with low-income levels [20], it is difficult for them to live in a high-quality school district. Therefore, compared with migrant workers, residents with local household registration are more likely to get higher quality educational resources.

The current literature mainly focuses on the impact of residence permits on public education services, and there are few studies on public health care services. Unlike public education services, most public health care services are not exclusive. Do residence permits improve the public health care services utilization by migrant workers? The answer to this question can help resolve the current debate about the effectiveness of residence permits and provide information to improve the residence permit. To provide evidence to achieve health equality between local urban and migrant workers in China, this study aims to investigate the effect of residence permits on the utilization of public health care services. We used a representative national survey to explore the following questions: (1) the specific characteristics of the utilization of public health care services of migrant workers; (2) whether the residence permit is sufficient enough to solve the health care access inequality faced by migrant workers; (3) whether the city size influences the validity of residence permit. Through a discussion of the above problems, we put forward some suggestions for improving the residence permit system.

## 2. Materials and Methods

### 2.1. Data Source

The data used in this paper came from the 2017 China Migrants Dynamic Survey (CMDS), which is the 10th consecutive cross-sectional survey conducted by the National Health Commission of China since 2009. CMDS covers 32 provincial units in China and conducts a sample survey of migrants aged over 15 years and living in the inflow area for more than one month. Based on the Probability Proportional to Size (PPS) approach, a stratified multi-stage random sampling method was used to take samples. The sampling process was divided into three steps by the PPS method. The first step was to select the township-level units in proportion among the 32 province-level administrative units. In the second stage, village committees were selected based on the size of the chosen townships. The third step was that the chosen village committees selected the migrants to investigate. The contents of the survey included information about the migrants and their families, employment status, public services, and social integration, which could meet the needs of this study.

Considering that our research objects were migrant workers, and most people retire at 60 in China, we selected samples according to the following conditions: (1) without local household registration; (2) in the state of employment or job hunting; (3) age at 15–60 years old; (4) no missing value. Ultimately, we got 128,757 respondents in this study. We also studied the impact of residence permits on the utilization of public health care services. In the survey, only respondents who had been ill in the past year answered questions about the utilization of public health care services. The definition of illness depends on the question of whether they had been ill (injured) or unwell in the last year. A total of 63,277 respondents answered “yes” to this question.

### 2.2. Measures

#### 2.2.1. Health Care Services Utilization

Health care is the maintenance or improvement of health via prevention, diagnosis, treatment, and recovery. In this paper, we chose public health services and medical services to measure health care services. Specifically, public health services mainly refer to the basic services that affect the risk factors of health and improve the level of disease prevention and control [39]. Medical services mainly refer to diagnosis, treatment, nursing, and rehabilitation services [40]. The former focus on prevention and control, while the latter focus on treatment and rehabilitation.

In China, public health services for all residents are divided into four categories, including the establishment of health records, health education, public health emergency management, and public health supervision. Considering the daily needs of migrant workers, we chose health records and health education as representatives of public health services. Specifically, health records depended on whether migrant workers had established health records (1 = yes, 0 = no), and health education depended on whether migrant workers had received health education (1 = yes, 0 = no).

For medical services, we chose hospital visits as an indicator to measure medical services utilization, which was a typical measurement [41,42]. In our study, the indicator was based on the question: “when you fall ill, which type of hospital would you choose first?” The choices of the answer were: “ignore”, “take medication”, “private clinic”, “primary hospital”, “general hospital”, “return to hometown”, and “other places”. Among these answers, private clinics in China are mainly concentrated at the village/community level [43], with inadequate medical facilities and few doctors. Primary hospitals and general hospitals both belong to the medical services provided by the government. In these two types of hospitals, migrant workers can be reimbursed for expenses through medical insurance. Compared with private clinics, hospitals have better medical facilities, more doctors, and higher quality health services [9]. According to the hierarchical medical system in China, the primary hospital’s medical service capacity was generally lower than that of the general hospital. Thus, we selected two independent variables, including hospital visits (1 = primary hospital or general hospital, 0 = others) and general hospital visits (1 = general hospital, 0 = others).

#### 2.2.2. Residence Permit

We took residence permit as the independent variable of theoretical interest. This variable was a binary variable based on the question “do you have a residence permit.” If the migrant workers had a residence permit, the variable would be assigned to 1, otherwise 0.

#### 2.2.3. Control Variables

According to Andersen’s behavior model, we controlled variables from three aspects: predisposing factors, enabling factors, and need factors [44]. Predisposing factors included gender (1 = male, 0 = female), age, education level, hukou (1 = rural hukou, 0 = urban hukou), and marital status (1 = married, 0 = unmarried). Among them, age depended on the age of migrant workers. Education level reflected the highest level of their educational background and was divided into five levels from low to high: primary school or below, junior high school, high school, college, graduate, or above. In China, due to the implementation of the household registration system, household registration is generally divided into rural hukou and urban hukou. Rural hukou refers to the residents registered in rural areas, while urban hukou is registered in urban areas. Enabling factors included income, work contract (whether they had signed a labor contract with the employer, 1 = yes, 0 = no), and homeownership (whether they had homeownership, 1 = yes, 0 = no). Income referred to the respondents’ average monthly income, which was divided into five categories: low income (below 3000 CNY), relatively low income (between 3000 CNY to 6000 CNY), middle income (between 6000 CNY to 10,000 CNY), relatively high income (between 10,000 CNY to 15,000 CNY), and high income (above 15,000 CNY). The signing of a labor contract means the stability of employment. Without the guarantee of a labor contract, the working status of migrant workers is precarious and vulnerable. Some migrant workers even receive daily wages in China, meaning that leaving work to seek health care will cause wage losses [21]. Need factors included self-reported health and chronic disease. Health status represented the self-reported health level of the migrant worker. Responses were given on a 4-point rating scale, ranging from 1 (very unhealthy) to 4 (very healthy). Chronic disease reflected whether the responder had hypertension or diabetes (1 = yes, 0 = no).

### 2.3. Estimation Method

After controlling for these variables described above, we first explored the effects of residence permits on the utilization of public health care services. The variables used to measure the utilization of public health care services in this paper were binary. Since the linear model might have heteroscedasticity and nonmorality when solving the binary variables, and the Probit model could better handle heteroscedasticity and non-normality [45], we used the Probit model for regression. The model was as follows:

(1) $$Y=\alpha +\beta X+\underset{j}{\sum }\gamma_{j}C_{j}+\lambda_{p}+\varepsilon$$

In this model, Y represented the dependent variable to measure the utilization of public health care services, X denoted the residence permit, $C_{j}$ represented the control variables, $\lambda_{p}$ represented the province-level fixed effects, ε denoted the error term. We used Stata 13.1 (Stata Corporation, College Station, TX, USA) for model regressions.

## 3. Results

### 3.1. Descriptive Analysis

Descriptive statistics are presented in Table 1. Only 29.5% of the migrant workers in the survey reported that they had established health records. The proportion of migrant workers who had accessed health education was 70.9%. A total of 35.4% of the respondents chose primary hospitals or general hospitals for medical treatment, while only 15.9% chose high-quality general hospitals.

In terms of the characteristics of the sampled migrant workers, the proportions of males and females were relatively balanced (56.5% were male). The average age was 35.973, the self-reported health status scored healthy on average, and only 4.2% of the respondents had chronic diseases, indicating that the sampled migrant workers were a middle-aged and healthy labor force. A total of 81.9% of the respondents were married. Regarding education level, 44.1% of the migrant workers had a junior high school education, and 22.2% had a high school education, indicating that they had generally received primary education. This was similar to other studies on the demographic characteristics of migrant workers [46,47]. As for income level, only 6.6% of the migrant workers belonged to the low-income category, meaning that most of the migrant workers’ income could meet daily life needs. Furthermore, 78.2% of the sample had rural hukou, indicating that most of them registered in rural areas. The homeownership rate was 27.7%, suggesting that most migrant workers did not have a stable living environment. Only 29.4% of migrant workers had signed a labor contract with the employer, indicating that most migrant workers did not have stable jobs and a stable income source.

Furthermore, we divided the sample into two groups: those with a residence permit and those without a residence permit. It could be found that compared with the migrant workers without a residence permit, those with residence permits had a higher proportion of establishing health records, receiving health education, and choosing hospital visits. The education background of these two groups was similar. In terms of income level, the overall level of migrant workers with residence permits was higher than that without a residence permit. Those with a residence permit had higher employment contract rates and lower homeownership rates than those without. Using an independent t-test, there were significant differences in most features between the migrants who had residence permits and those who did not (*p* < 0.01).

### 3.2. Utilization of Public Health Services

Table 2 presents regression results of factors related to the utilization of public health services. For each variable, we reported the average marginal effects. We first tested the impacts of the residence permit on the health record and health education. After controlling for all individual characteristics and province effects, the marginal effect of residence permit was positive and statistically significant, indicating that migrant workers with residence permits were more likely to use public health services than other migrant workers. Specifically, those with residence permits had a higher probability to establish health records and receive health education, 7.9% and 6.5%, respectively. Furthermore, with the improvement of educational background, the probabilities of migrant workers establishing health records and receiving health education increased gradually. It was worth noting that compared with low-income groups, high-income groups were less likely to establish health records and receive health education.

### 3.3. Utilization of Public Medical Services

Table 3 presents regression results of factors related to the utilization of public medical services. We found that holding a residence permit significantly increased the likelihood of migrant workers going to the hospital after falling ill. Migrant workers with residence permits were 4.6% more likely to see a doctor in hospitals than other migrant workers, and 1.2% more likely to be in general hospitals. With improvements in education level of migrant workers, the positive impacts on the utilization of public medical services gradually expanded. Taking hospital visits as an example, the positive effects of education level increased from 2.4% (junior high school) to 4.1% (high school), then to 5.6% (college), and finally to 6.0% (graduate and above). Furthermore, the increase of education level improved the probability of migrant workers seeking higher quality medical services. The positive impacts of junior high school and high school on hospital visits (2.4% and 4.1%, respectively) were higher than that of general hospital visits (2.2% and 3.9%, respectively). On the contrary, the positive impact of college and graduate and above education on hospital visits (5.6% and 6.0%, respectively) was lower than that on general hospital visits (6.1% and 7.7%, respectively). We also found that for groups with relatively high income and high income, their positive impacts on general hospital visits (3.2% and 6.6%, respectively) were much higher than that on hospital visits (1.6% and 2.8%, respectively), indicating that migrant workers with high income had higher probabilities of seeking higher quality medical services.

### 3.4. Utilization in Megacities

In Table 4, we examined the moderating effect of city size on residence permits and utilization of health care services to explore the utility of residence permit in megacities. We defined megacities as cities with a population of more than 10 million. In our sample, a total of 12 cities were megacities, namely Shenzhen, Beijing, Hangzhou, Zhengzhou, Wuhan, Chongqing, Tianjin, Shanghai, Nanjing, Shenyang, Chengdu, and Guangzhou. These cities are located in multiple areas across China, making the results representative. Surprisingly, we found that the moderating effects of megacities on health record, health education, and hospital visits were statistically significant and negative, indicating that the effect of residence permit on the utilization of health care services in megacities was even lower than the national level.

## 4. Discussion

Using nationally representative data in China, we examined the association of residence permits on the utilization of health care services by migrant workers from two aspects: public health services and public medical services. The possession of residence permits significantly improved the probability of migrant workers using public health services and public medical services, indicating that the role played by residence permits was effective. However, the effects of residence permits were limited. In megacities, the positive effects of residence permits on health care service utilization of migrant workers were even lower than the overall level.

The residence permit is an important certificate to ensure that migrant workers enjoy the same rights as urban locals in all aspects. We found that only 67.9% of respondents had residence permits, which was far from the goal of universal coverage. The coverage of residence permits varied in different provinces. The provinces with a higher penetration rate of residence permits were concentrated in eastern China, which is also the largest labor force input place for migrant workers. In 2017, nearly 160 million migrant workers chose to work in eastern China, accounting for 55.8% of the total number [48]. The influx of migrant workers has brought sufficient labor to support regional development and caused great pressure on immigration management [49], which has prompted provinces in eastern China to put more effort into this work. Issuing residence permits to migrant workers is conducive to guarantee their rights to use public services and value to administrators in the management of migrants. Especially in the outbreak of serious infectious diseases, tracking migrant workers who may be infected and their contacts is tough because some are not registered with the local government and have little formal contact with mainstream society [50]. The regional coverage of residence permits solves this problem, putting the Chinese government more at ease during the COVID-19 epidemic than during SARS. However, the coverage of residence permits in most areas of central and western China still lags behind, which requires the attention of policymakers.

The results showed that migrant workers did not make full use of public health services. In terms of the utilization of public health services, the popularization of health records remains to be improved. Our research showed that only 29.5% of migrant workers had established health records, whereas the national average was 76.4% [51]. Health records contain rich clinical information [52], which significantly shortens the time and improves the quality of medical consultations. Establishing health records for migrant workers is challenging because of the restrictions of the household registration system [53]. Our study also found that 70.9% of the respondents had received health education. At present, strengthening health literacy through health education has become a common suggestion in previous research [54,55]. Since online education is more convenient, less expensive, and more effective than offline education [21], online education could be the main way to improve health education for migrant workers, supplemented by offline education. In addition, the utilization of public medical services by migrant workers was insufficient. Only 35.4% and 15.9% of migrant workers chose hospital visits and general hospital visits after they got sick, respectively. Untreated illness might affect their daily work and life [41]. Therefore, health education for migrant workers should focus on timely treatment after illness to improve medical utilization.

Regarding the validity of residence permits, many researchers believe that the household registration system’s reform is critical to solving health care access inequality [47,56,57]. However, our results showed that residence permits, which aim to improve restrictions of the household registration system, did not address health inequalities faced by migrant workers tremendously. Compared with the great health care access inequalities faced by migrant workers, residence permits’ positive impact was limited. Although migrant workers with residence permits were 7.9% more likely to establish health records, the residence permit’s impact was muted. The substantive significance is modest, even if it is statistically significant. Similarly, the impact of residence permits on the utilization of public medical services was significant but limited.

Furthermore, in exploring the impact of city size on the validity of residence permits, we found that the impact of residence permits on the utilization of health care services in megacities was lower than the overall level. This might be because local governments in megacities tend to favor only high-skilled people rather than opening up to low-skilled people. Due to the limitation of urban comprehensive carrying capacity in China’s megacities, a points-based system is implemented for the residence permit. The score of the points directly corresponds to the quantity of basic public services obtained by the residence permit holder. Before obtaining certain points, there are always differences between the residence permit holders’ basic public service rights and the local residents. With limited education levels, migrant workers are often unable to get higher scores and obtain the same rights to basic public services as local residents. In our research sample, 78.2% of migrant workers were from rural areas with insufficient educational resources, and most of them could only engage in low-skilled jobs. The development of cities needs the full spectrum of high-grade investment migrants, high-skilled migrants, and relatively low-skilled labor migrants. Employment opportunities are provided by the market, and public resources are provided by the government. While attracting more labor forces, urban policymakers should also provide some public resources for low-skilled migrant workers so that the residence permit can benefit all levels of migrants.

Undeniably, there are still some problems in the residence permit system, which need to be further improved. However, the factors affecting the utilization of public health care services by migrant workers are also related to their precarious status [21], including income, career stability, gender, age, etc. [2,57,58]. Taking income as an example, as Peng et al. (2010) pointed out, the main reason for restricting the utilization of medical services by migrant workers was insufficient affordability [59]. The high costs of health care lead migrants to use unsupervised self-treatment or substandard care [28,57], which puts them at risk of worse health outcomes than local residents [30]. Migrant workers’ average wage is naturally lower than that of urban locals [20]. The income gap of migrant workers is partly due to wage inequality caused by household registration restrictions [60]. However, more importantly, due to the low level of education, many migrant workers are engaged in low-skilled jobs [61], indicating that they not only had low income in the current period, but also had limited space to obtain more income and were more easily replaced. Furthermore, according to our research results, the labor contract signing rate of migrant workers was only 29.4%, and even for those who had got residence permits, the signing rate was only 30.0%, which was far lower than the national average of 90% reported by the Chinese government [48]. Future policies need to improve the labor contract signing rate of migrant workers to ensure stable employment. The reason for the fragile status of migrant workers lies in not only household registration restrictions, but also the urban–rural development gap caused by the urban–rural dualistic economic structure and the joint influence of other factors. Therefore, health care access inequality faced by migrant workers is a structural problem. The restriction of a household registration system is only one of the causes of health care access inequality. While ensuring the equal rights of migrant workers and the local people, the local government should also strengthen the vocational skills training of migrant workers to reduce the high substitutability caused by low-skilled jobs as far as possible so that they can better integrate into mainstream society.

This study has several limitations. First, there were differences in the contents of the points system and residence permit system among different provinces or cities, which might lead to different system effects. To avoid the influence caused by the differences, we controlled for provincial fixed effects in the analysis. Future studies can classify different provinces or cities according to the contents of the residence permit system, to explore the different categories of provinces and more accurately understand the effect of the residence permit system. Second, this study’s health resource utilization focused on the sickness treatment that migrant workers chose first in the last illness, which is insufficient to measure the utilization. In follow-up research, we can use the health records of health care facilities to analyze the utilization of health care resources and provide more information to solve health care access inequality. Third, subject to data restrictions, we did not distinguish between the severity of migrant workers’ diseases. The difference in disease severity might directly affect the medical choice of migrant workers. Although we alleviated this problem by controlling for the health status of migrant workers, it would be more valuable to distinguish the severity of the disease. Fourth, due to data limitations, there was a lack of further discussion on the subjective reasons for migrant workers to apply for residence permits. In the future, we will conduct surveys and collect data from this perspective. Fifth, the data in this paper cannot be directly compared with the data of local residents, but the problem of insufficient utilization of public health services by migrant workers can be indirectly illustrated by reference to the literature. We compared the results of the study with the national average level through literature citations in this study. In the future, we will further investigate the differences between migrant workers and local residents to explore more accurate data.

## 5. Conclusions

The residence permits issued by the Chinese government aim to give migrant workers the same rights as urban residents, helping them make better utilization of public services and promoting health equality. However, there has been controversy about the actual effect of residence permits. In this paper, we used a representative national survey to examine the impact of residence permits on public health care services. Our results showed that migrant workers with residence permits were significantly more likely than other migrant workers to utilize public health care services, which provided empirical support for the positive effect of residence permits. However, the effect of residence permits was limited. Megacities had significant negative moderating effects between residence permits and health care service utilization. This showed that the current residence permit system still needs to be improved. For developing countries with uneven regional development, health care access inequality faced by migrant workers is a structural problem, and limitations of verification identity are just one of the causes. We hope that our research can bring inspiration to future researchers in health care access inequality and provide some references for countries with health care access inequality.

## Figures and Tables

**Table 1:** Descriptive statistics of variables.

Variable	Overall Mean	CI 95%	Group Mean		Independent *t*-Test	
			With RP	Without RP	Difference	*p*-Value
Health record	0.295	(0.292. 0.297)	0.305	0.273	0.032	0.000
Health education	0.709	(0.706, 0.711)	0.717	0.692	0.024	0.000
Hospital visit	0.354	(0.350, 0.358)	0.375	0.312	0.063	0.000
General hospital visit	0.159	(0.156, 0.162)	0.165	0.146	0.019	0.000
Residence permit	0.679	(0.676, 0.681)	—	—	—	—
Gender	0.565	(0.563, 0.568)	0.577	0.541	0.035	0.000
Age	35.973	(35.923, 36.024)	36.281	35.325	0.955	0.000
Primary school and below	0.150	(0.149, 0.152)	0.153	0.146	0.007	0.001
Junior high school	0.441	(0.438, 0.444)	0.451	0.420	0.032	0.000
High school	0.222	(0.220, 0.224)	0.221	0.224	−0.004	0.132
College	0.110	(0.108, 0.111)	0.103	0.124	−0.021	0.000
Graduate and above	0.077	(0.076, 0.078)	0.073	0.087	−0.014	0.000
Hukou	0.782	(0.780, 0.784)	0.786	0.773	0.014	0.000
Marital status	0.819	(0.816, 0.821)	0.837	0.780	0.057	0.000
Low income	0.066	(0.064, 0.067)	0.053	0.093	−0.040	0.000
Relatively low income	0.383	(0.380, 0.386)	0.366	0.420	−0.054	0.000
Middle income	0.336	(0.333, 0.339)	0.345	0.317	0.029	0.000
Relatively high income	0.133	(0.131, 0.135)	0.141	0.116	0.025	0.000
High income	0.082	(0.081, 0.084)	0.095	0.055	0.041	0.000
Work contract	0.294	(0.292, 0.297)	0.300	0.282	0.018	0.000
Home ownership	0.277	(0.274, 0.279)	0.250	0.333	−0.083	0.000
Self-reported health	3.829	(3.827, 3.831)	3.835	3.816	0.019	0.000
Chronic disease	0.042	(0.041, 0.044)	0.042	0.043	0.000	0.828

Note: CI refers to confidence interval. With RP means migrant workers with residence permit, Without RP otherwise.

**Table 2:** Average marginal effects of residence permit on the utilization of public health services.

Variables	Health Record			Health Education		
	A.M.E	95% CI	*p*-Value	A.M.E	95% CI	*p*-Value
Residence permit	0.079	(0.074, 0.085)	0.000	0.065	(0.059, 0.070)	0.000
Gender	−0.025	(−0.030, −0.020)	0.000	−0.021	(−0.026, −0.016)	0.000
Age	0.000	(0.000, 0.001)	0.011	−0.001	(−0.001, −0.001)	0.000
Junior high school	0.025	(0.018, 0.033)	0.000	0.061	(0.053, 0.069)	0.000
High school	0.038	(0.030, 0.047)	0.000	0.090	(0.081, 0.099)	0.000
College	0.055	(0.044, 0.065)	0.000	0.086	(0.075, 0.097)	0.000
Graduate and above	0.063	(0.050, 0.075)	0.000	0.102	(0.089, 0.114)	0.000
Hukou	−0.018	(−0.024, −0.011)	0.000	−0.019	(−0.026, −0.012)	0.000
Marital status	0.041	(0.034, 0.048)	0.000	0.059	(0.052, 0.066)	0.000
Relatively low income	0.000	(−0.011, 0.010)	0.933	0.018	(0.008, 0.029)	0.000
Middle income	−0.005	(−0.016, 0.006)	0.370	0.015	(0.004, 0.026)	0.007
Relatively high income	−0.019	(−0.031, −0.007)	0.002	−0.002	(−0.014, 0.010)	0.720
High income	−0.026	(−0.039, −0.012)	0.000	−0.016	(−0.029, −0.002)	0.027
Work contract	0.056	(0.050, 0.061)	0.000	0.051	(0.045, 0.057)	0.000
Home ownership	0.038	(0.032, 0.043)	0.000	0.010	(0.004, 0.015)	0.002
Self-reported health	0.047	(0.041, 0.053)	0.000	0.031	(0.025, 0.037)	0.000
Chronic disease	0.021	(0.008, 0.033)	0.001	−0.002	(−0.014, 0.010)	0.751
Provincial fixed effect	Yes			Yes		
Observations	128,757			128,757		

Note: A.M.E refers to average marginal effect. CI refers to confidence interval. The reference of education level is primary school and below and the reference of income level is low income.

**Table 3:** Average marginal effects of residence permit on the utilization of public medical services.

Variables	Hospital Visit			General Hospital Visit		
	A.M.E	95% CI	*p*-Value	A.M.E	95% CI	*p*-Value
Residence permit	0.046	(0.038, 0.055)	0.000	0.012	(0.006, 0.019)	0.000
Gender	−0.018	(−0.025, −0.010)	0.000	−0.017	(−0.023, −0.011)	0.000
Age	−0.001	(−0.001, 0.000)	0.000	−0.001	(−0.001, 0.000)	0.002
Junior high school	0.024	(0.013, 0.036)	0.000	0.022	(0.013, 0.030)	0.000
High school	0.041	(0.028, 0.054)	0.000	0.039	(0.029, 0.049)	0.000
College	0.056	(0.039, 0.073)	0.000	0.061	(0.049, 0.074)	0.000
Graduate and above	0.060	(0.041, 0.079)	0.000	0.077	(0.062, 0.092)	0.000
Hukou	0.001	(−0.009, 0.011)	0.838	−0.006	(−0.014, 0.001)	0.112
Marital status	0.015	(0.003, 0.026)	0.011	0.007	(−0.002, 0.015)	0.137
Relatively low income	0.002	(−0.015, 0.018)	0.850	0.012	(0.000, 0.024)	0.052
Middle income	0.010	(−0.007, 0.027)	0.236	0.015	(0.003, 0.028)	0.019
Relatively high income	0.016	(−0.003, 0.035)	0.099	0.032	(0.018, 0.046)	0.000
High income	0.028	(0.007, 0.049)	0.009	0.066	(0.050, 0.082)	0.000
Work contract	0.040	(0.031, 0.049)	0.000	0.012	(0.006, 0.019)	0.000
Home ownership	0.026	(0.017, 0.034)	0.000	0.027	(0.020, 0.034)	0.000
Self-reported health	−0.052	(−0.060, −0.044)	0.000	−0.062	(−0.068, −0.056)	0.000
Chronic disease	0.058	(0.041, 0.075)	0.000	0.036	(0.023, 0.048)	0.000
Provincial fixed effect	Yes			Yes		
Observations	63,277			63,277		

Note: A.M.E refers to average marginal effect. CI refers to confidence interval. The reference of education level is primary school and below and the reference of income level is low income.

**Table 4:** Moderating effect of city size between residence permit and the utilization of health care services.

Variables	Health Record			Health Education			Hospital Visit			General Hospital Visit		
	A.M.E	95% CI	*p*-Value	A.M.E	95% CI	*p*-Value	A.M.E	95% CI	*p*-Value	A.M.E	95% CI	*p*-Value
Residence permit	0.082	(0.076, 0.088)	0.000	0.075	(0.069, 0.081)	0.000	0.052	(0.042, 0.061)	0.000	0.014	(0.007, 0.021)	0.000
Residence permit × Megacity	−0.014	(−0.024, −0.005)	0.002	−0.041	(−0.050, −0.032)	0.000	−0.021	(−0.034, −0.007)	0.002	−0.005	(−0.015, 0.005)	0.325
Gender	−0.025	(−0.030, −0.020)	0.000	−0.021	(−0.026, −0.016)	0.000	−0.018	(−0.025, −0.010)	0.000	−0.017	(−0.023, −0.011)	0.000
Age	0.000	(0.000, 0.001)	0.010	−0.001	(−0.001, −0.001)	0.000	−0.001	(−0.001, 0.000)	0.000	−0.001	(−0.001, 0.000)	0.002
Junior high school	0.025	(0.018, 0.033)	0.000	0.062	(0.054, 0.069)	0.000	0.025	(0.013, 0.036)	0.000	0.022	(0.013, 0.030)	0.000
High school	0.038	(0.030, 0.047)	0.000	0.091	(0.082, 0.100)	0.000	0.042	(0.028, 0.055)	0.000	0.039	(0.029, 0.049)	0.000
College	0.055	(0.045, 0.066)	0.000	0.088	(0.077, 0.099)	0.000	0.057	(0.041, 0.074)	0.000	0.062	(0.049, 0.074)	0.000
Graduate and above	0.064	(0.051, 0.076)	0.000	0.105	(0.092, 0.117)	0.000	0.061	(0.042, 0.081)	0.000	0.077	(0.062, 0.093)	0.000
Hukou	−0.018	(−0.024, −0.011)	0.000	−0.019	(−0.026, −0.013)	0.000	0.001	(−0.009, 0.011)	0.852	−0.006	(−0.014, 0.001)	0.111
Marital status	0.041	(0.034, 0.048)	0.000	0.059	(0.052, 0.066)	0.000	0.015	(0.003, 0.026)	0.012	0.007	(−0.002, 0.015)	0.138
Relatively low income	0.000	(−0.011, 0.010)	0.948	0.019	(0.008, 0.029)	0.000	0.002	(−0.015, 0.018)	0.843	0.012	(0.000, 0.024)	0.052
Middle income	−0.005	(−0.015, 0.006)	0.397	0.015	(0.005, 0.026)	0.005	0.011	(−0.006, 0.028)	0.223	0.015	(0.003, 0.028)	0.018
Relatively high income	−0.018	(−0.030, −0.006)	0.003	−0.001	(−0.013, 0.011)	0.873	0.016	(−0.002, 0.035)	0.086	0.032	(0.018, 0.046)	0.000
High income	−0.025	(−0.038, −0.011)	0.000	−0.013	(−0.027, 0.001)	0.068	0.029	(0.008, 0.050)	0.006	0.067	(0.050, 0.083)	0.000
Work contract	0.056	(0.050, 0.061)	0.000	0.051	(0.045, 0.056)	0.000	0.040	(0.031, 0.049)	0.000	0.012	(0.006, 0.019)	0.000
Home ownership	0.038	(0.032, 0.043)	0.000	0.010	(0.004, 0.015)	0.002	0.025	(0.017, 0.034)	0.000	0.027	(0.020, 0.034)	0.000
Self-reported health	0.047	(0.041, 0.053)	0.000	0.031	(0.025, 0.037)	0.000	−0.052	(−0.060, −0.044)	0.000	−0.062	(−0.068, −0.056)	0.000
Chronic disease	0.021	(0.008, 0.033)	0.001	−0.002	(−0.014, 0.010)	0.752	0.058	(0.041, 0.075)	0.000	0.036	(0.023, 0.048)	0.000
Provincial fixed effect	Yes			Yes			Yes			Yes		
Observations	128,757			128,757			63,277			63,277		

Note: A.M.E refers to average marginal effect. CI refers to confidence interval. The reference of education level is primary school and below and the reference of income level is low income.

## Data Availability

Restrictions apply to the availability of these data. Data were obtained from the Migrant Population Service Center, National Health Commission China and are available online at http://www.chinaldrk.org.cn (accessed on 6 May 2021) with the permission of Migrant Population Service Center, National Health Commission China.
